# Egg-level temperature monitoring and CFD-guided structural optimization improve thermal uniformity and hatch performance in a single-stage tunnel incubator

**DOI:** 10.1016/j.psj.2026.106666

**Published:** 2026-02-18

**Authors:** Mingyang Li, Zefeng Shi, Zongchun Bai, Yinong Hu, Guofeng Han

**Affiliations:** a Institute of Agricultural Facilities and Equipment, Jiangsu Academy of Agricultural Sciences, Nanjing 210014, China; b Key Laboratory of Protected Agriculture Engineering in the Middle and Lower Reaches of Yangtze River, Ministry of Agriculture and Rural Affairs, Nanjing 210014, China

**Keywords:** Tunnel incubator, Eggshell temperature, Chick quality, Computational fluid dynamics, Porous-medium model

## Abstract

Temperature uniformity inside commercial tunnel incubators is critical for hatchability and chick quality, but quantitative field data and validated engineering solutions remain limited. This study combined in situ EST measurements, hatch performance evaluation and computational fluid dynamics (CFD) modeling to characterize and improve the thermal environment in a single-stage tunnel incubator. In one tunnel incubator (capacity 90,720 eggs; operated under standard single-stage conditions), eggshell temperature (EST) was monitored at 15 locations (5 trolleys × 3 vertical tiers; 1-min logging for 12 h on embryonic day 1) during half-capacity (37,800 eggs) loading. Hatchability, hatch time, and chick weight uniformity were evaluated by tier using 5 replicate hatches, with trolley-within-tier as the experimental unit. A porous-medium CFD model was validated against the 15-point EST dataset and used to test transverse trolley orientation and a perforated flow-straightening plate. Under baseline conditions, maximum intra-trolley temperature differences reached 0.84°C and trolley-level non-uniformity indices approached 0.62±0.33%. Eggs on the middle tier, which experienced warmer and more stable temperatures, showed higher hatchability (*p* < 0.05), earlier mean hatch time (*p* < 0.05) and greater chick weight uniformity (*p* < 0.05) than upper and lower tiers. The CFD model reproduced measured temperatures with a maximum absolute deviation of 0.35°C and predicted that transverse trolley orientation could reduce non-uniformity indices to 0.29±0.12%, while the perforated plate reduced them to 0.38±0.14%. Field implementation of transverse trolley orientation confirmed improved temperature uniformity. These findings demonstrate that egg-level monitoring combined with CFD-guided structural optimization can substantially improve the thermal environment in commercial tunnel incubators and support more uniform broiler chick production.

## Graphical abstract

**Figure:**
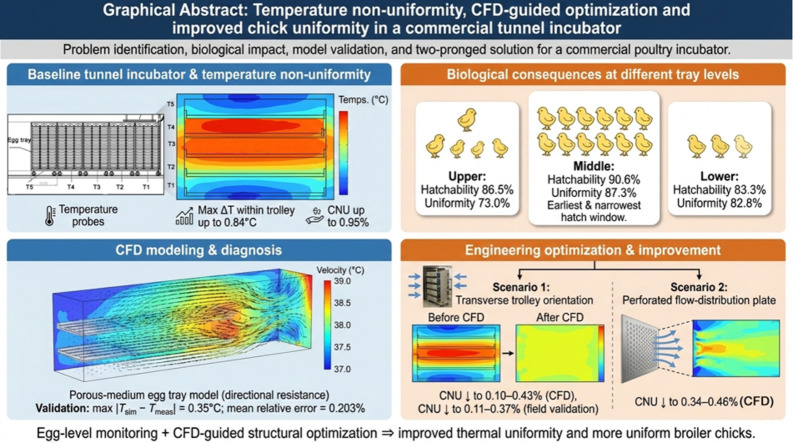

## Introduction

Efficient incubation is fundamental to the profitability and sustainability of the broiler industry. In modern hatcheries, millions of chicks are produced weekly, and relatively small changes in hatchability, hatch window or chick uniformity can result in into substantial economic gains or losses. The physical environment inside incubators, particularly air temperature and its spatial and temporal uniformity around the eggs, is a key determinant of embryonic development, hatch success and subsequent broiler performance (Ipek et al., 2014; Wijnen et al., 2020; Yalcin et al., 2022). Even when incubators operate at the correct set-point temperature, local deviations at the egg level can impair embryogenesis, expands the spread of hatch, increase late embryo mortality and produce chicks of inferior and uneven quality (Boerjan, 2006; Bergoug et al., 2013).

Commercial tunnel incubators are widely used in large-scale broiler hatcheries because they offer high capacity and efficient use of floor space. In these systems, multiple egg trolleys are arranged in series along an air distribution tunnel, and conditioned air is driven through the egg stacks by axial fans. However, the large volume, complex geometry and high egg density of tunnel incubators make it challenging to maintain a uniform thermal environment throughout the machine. Previous studies have reported significant spatial variations in air velocity and temperature within single-stage or multi-stage incubators and have linked these variations to differences in hatchability and chick quality (Lourens et al., 2011; Nowaczewski et al., 2014; Seok et al., 2025). Most of this work, however, has focused on cabinet-type incubators or has been conducted under experimental conditions with limited egg numbers. Quantitative field data describing temperature non-uniformity and its consequences for hatch performance in commercial tunnel incubators remain scarce.

Computational fluid dynamics (CFD) has become a powerful tool for analyzing airflow and heat transfer in animal production facilities, including poultry houses and incubators (Durodola, 2023; Fernandes, 2024). By resolving coupled three-dimensional airflow and heat-transfer fields, CFD can localize regions of low ventilation effectiveness and thermal non-uniformity within setters/hatchers and can be used to virtually test design or operational changes before implementation (Eren Ozcan et al., 2010; Seok et al., 2025). However, incubator CFD studies frequently rely on simplified geometries and boundary conditions (e.g., porous-medium representations of egg trays or racks), and therefore require rigorous validation against spatially dense measurements collected in operating commercial machines to ensure predictive accuracy (Kü üktop U et al., 2024). Moreover, only a limited body of work has explicitly progressed from CFD-based optimization to field-implementable modifications and then quantified benefits using hatchery-relevant biological outcomes such as hatchability, hatch spread (hatch window), and chick quality or uniformity (Seok et al., 2025). In commercial systems, egg position within the incubator (among trolleys and across vertical tiers) can generate systematic differences in eggshell temperature (EST) due to airflow maldistribution and buoyancy effects associated with embryonic metabolic heat production (French, 1997; Elibol and Brake, 2008). Because relatively small shifts in EST can alter embryonic development, shift hatch completion time, and compromise chick quality, there is a practical need to quantify trolley- and tier-specific thermal gradients under commercial operating conditions and to evaluate simple, low-cost airflow interventions that can be retrofitted into existing tunnel incubators (Lourens et al., 2005; Ipek et al., 2014; Yalcin et al., 2022).

Therefore, the objectives of the present study were to: (i) characterize the spatial distribution of EST and quantify trolley-level temperature non-uniformity in a commercial tunnel incubator; (ii) evaluate the effects of vertical trolley position (top, middle and bottom tiers) on hatchability, mean hatch time and chick weight uniformity; (iii) develop and validate a CFD model of the tunnel incubator using a porous-medium representation of the egg trays and eggs; (iv) use the validated model to assess two structural optimization scenarios—transverse trolley orientation and installation of a perforated flow-straightening plate; and (v) experimentally verify the most promising optimization scenario under commercial hatchery conditions. We hypothesized that pronounced vertical thermal gradients exist within the tunnel incubator, that eggs located on the middle tier achieve superior hatch performance and uniformity compared with those on the top and bottom tiers, and that relatively simple structural modifications can substantially reduce temperature non-uniformity and thereby improve the thermal environment experienced by the eggs.

## Materials and methods

### Commercial hatchery, birds and incubator

This study was performed according to the Research Committee of the Jiangsu Academy of Agricultural Sciences and was carried out in strict accordance with the Regulations for the Administration of Affairs Concerning Experimental Animals (Permit Number SYXK (Su) 2020-0024).

Fertile broiler hatching eggs were obtained from a single commercial breeder flock (Huashan Yellow Chicken line 4; flock age 45 wk) managed under standard breeder conditions. Eggs (E12) were collected, stored at 18°C and 50% RH before setting, and graded according to the hatchery’s routine quality criteria (no cracks, normal shape and shell quality).

**Fig. 1:**
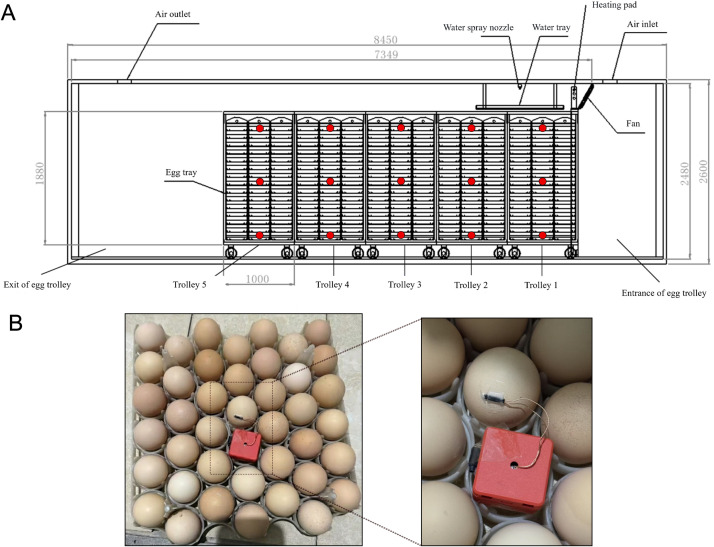
**Schematic of the incubator layout with the specific sensor locations.** Schematic diagram of the single-stage tunnel incubator used in this study. (A) Five egg trolleys (T1–T5) are arranged along the longitudinal airflow direction, numbered from the air inlet (T1) to the air outlet (T5). The air-handling unit, fans, heater, and air channel are indicated. Circles mark the positions of eggshell temperature data loggers on the upper, middle, and lower tray levels of the second tray row on each trolley. A total of 15 temperature measurement points (red plot) were established. (B) The specific sensor locations on eggs.

The egg trolley (model EIFXDZ-90720, Qingdao, China) measured 1250 mm (L) × 1000 mm (W) × 2080 mm (H) and was constructed entirely from steel plates. It contained three rows (front-to-back), with four egg trays per row. Each tray held 42 hatching eggs. The trolley comprised 15 vertical tiers, with a maximum capacity of 7,560 eggs per trolley. For EST mapping, 15 eggs (one per tier × trolley) were instrumented. For hatchability and chick quality, five replicate hatches were conducted; within each hatch, 15 tiers within-trolley were monitored, and each trier constituted one experimental unit (Fig. 1A). The incubator and hatcher were operated according to the hatchery’s standard broiler program, with set-point air temperatures and relative humidity levels adjusted by stage of incubation to achieve an EST close to 37.8°C. Trolley were turned at 90°, after which turning ceased at transfer. Ventilation was provided by 4 axial fans (manufacturer Qingdao Xingyi Electronic Equipment Co., Ltd., China), with 100% recirculation and CO2-dependent fresh-air inlet control according to the hatchery program. After transfer to the hatcher, temperature and RH were maintained at 36.8-37.2°C and 50-65% RH (set-point), respectively.

### Temperature measurements in the tunnel incubator

To characterize the spatial distribution of the thermal environment in the tunnel incubator, EST was monitored at 15 locations under set incubation temperature. One temperature sensor was mounted on the surface of a representative egg in the central region of each tier (top, middle and bottom) on each of the five trolleys (T1–T5), as illustrated in Fig. 1A. Sensors were positioned at mid-depth of the egg tray stack to minimize edge effects (Fig. 1B).

EST was measured using type T thermocouples (FLUKE, 80PK-1, USA) with an accuracy of ±1.1°C within the range 35–85°C. Sensors were secured to the eggshell using thermally conductive adhesive tape (3 M, 8810, USA), ensuring good thermal contact while avoiding interference with tray loading or air circulation. Temperature data were recorded at 1-min intervals over a 12-h period during the middle stage of incubation, when embryonic heat production is substantial and thermal control is most challenging.

For each measurement location, temperature recordings over the 12-h period were averaged to obtain a mean EST. For each trolley, the maximum temperature difference within that trolley (ΔT_max) was calculated as (1-1):

(1-1) $$\Delta T_{\text{max}}=\text{max}\left(T_{\text{top}},T_{\text{middle}},T_{\text{bottom}}\right)-\text{min}\left(T_{\text{top}},T_{\text{middle}},T_{\text{bottom}}\right)$$

where $T_{\text{top}}$, $T_{\text{middle}}$and $T_{\text{bottom}}$denote the mean EST at the top, middle and bottom tiers of the trolley, respectively. The trolley-level temperature non-uniformity index (CNU, %) was then computed as (1-2):

(1-2) $$\text{CNU}=\frac{\Delta T_{\text{max}}}{\overset{ˉ}{T}}\times \;100\%$$

where $\overset{ˉ}{T}$is the mean EST averaged across the three tiers of the trolley. These indices are summarized in Table 1.

**Table 1:** Eggshell temperature parameters of each trolley in the baseline configuration (measured).

Trolley	Max ΔT (°C)	Mean T (°C)	CNU (%)
T1	0.52	37.35	0.62
T2	0.55	37.80	0.64
T3	0.52	37.79	0.63
T4	0.84	37.72	0.95
T5	0.57	37.57	0.63

Max ΔT (°C), Maximum temperature difference(°C) Mean T (°C), Mean temperature(°C) CNU, coefficient of non-uniformity.

### Hatchability trial and chick measurements

To evaluate the effects of vertical trolley position on hatch performance and chick quality, eggs were set such that the top, middle and bottom tiers of the tunnel incubator could be compared under otherwise identical conditions. For this purpose, Five trolleys per tier were designated as experimental replicates. Each trolley was loaded to full commercial capacity with broiler hatching eggs from the same breeder flock and storage batch.

After completion of the setting phase in the tunnel incubator, eggs from all tiers were transferred to the same hatcher model. During transfer, eggs originating from the top, middle and bottom tiers of each trolley were placed into separate hatcher trays and carefully labelled to preserve information on their original vertical position. The hatcher was operated under the hatchery’s standard conditions until pull time.

At transfer, all eggs in the trial were candled to remove clear (infertile) eggs and early dead embryos, and only fertile eggs were included in subsequent calculations. Hatchability of fertile eggs (%) was calculated for each tier and trolley as (2-1):

(2-1) $$\text{Hatchability}=\frac{\text{Number}\;\text{of}\;\text{chicks}\;\text{hatched}}{\text{Number}\;\text{of}\;\text{fertile}\;\text{eggs}\;\text{set}}\times \;100\%$$

Mean hatch time was determined for each trolley and tier as the average time from setting in the tunnel incubator to chick emergence. Hatch times were recorded by continuous video (Yoosee camera, IPC09-1, 1080 p resolution, 60 fps, China) monitoring with time-lapse analysis (Romanini et al., 2013; Bergoug et al., 2015). For each trolley and tier, the hatch window (spread of hatch) was calculated as the difference between the hatch time of the last chick and that of the first chick within the same experimental unit, a standard definition used in incubation research (Zhong et al., 2018).

At pull time, all live chicks from each tier and trolley were individually weighed using an electronic scale (Deli, TE900, accuracy of 0.01 kg). Chick weight uniformity was expressed as the proportion of chicks whose body weight fell within ±10% of the mean body weight of that group (tier × trolley). This proportion was used as an index of chick weight uniformity at hatch.

### CFD model development

A three-dimensional CFD model of the tunnel incubator was developed to analyze airflow and heat transfer and to evaluate structural optimization scenarios. The computational domain included the main air distribution tunnel, the five egg trolleys and the surrounding walls. Geometric features were based on manufacturer specifications and field measurements of the commercial incubator.

To reduce computational cost while preserving the essential flow and thermal characteristics, the egg trays and eggs on each trolley were represented as a homogeneous porous medium (Fig. S1). The porous region was assigned appropriate porosity and directional flow resistance coefficients to reflect the pressure drop and flow distribution through the egg stacks. Viscous and inertial resistance terms were defined according to Darcy-Forchheimer equation (Fares et al., 2022) (3-1):

(3-1) $$\frac{\Delta P}{\Delta n}=-\left(D\mu \nu_{n}+C\frac{1}{2}\rho \left|\nu \right|\nu_{n}\right)$$

P is the pressure of the fluid, Pa; n is the distance of movement in the direction of fluid motion, m; μ is the dynamic viscosity, Pa · s; D is the coefficient of viscous resistance, m^-2^;C is the coefficient of inertia drag, m^-1^; *ρ* is the fluid density, kg/m^3^; ν is the velocity vector in the direction of fluid motion, m/s.

The governing equations for steady-state, incompressible airflow and heat transfer (continuity, Reynolds-averaged Navier–Stokes and energy equations) were solved using ANSYS Fluent, version 2025R1; ANSYS Inc., Canonsburg, PA, USA. Turbulence was modelled with the realizable k–ε model with standard wall functions (Shih et al., 1995). Air was treated as an ideal gas with temperature-dependent properties. Egg stacks were treated as a porous solid region with an effective thermal conductivity representing the combined effects of eggs, trays and interstitial air. Internal heat generation by embryos during the measurement stage was represented as a uniform volumetric heat source term calibrated against measured EST (Seok et al., 2025).

A structured or hybrid unstructured mesh was generated for the computational domain, with local refinement in regions of high velocity gradients (near fans, trolleys and the flow-straightening devices in optimization scenarios). Grid independence was checked by successively refining the mesh until changes in predicted mean ESTs at monitoring locations were less than 0.1°C. Convergence was assumed when all residuals decreased below 10⁻⁵ and when mass and energy balances closed within 1%. See detail in supplement materials(Celik et al., 2008).

### Model validation

The CFD model was validated against the EST measurements obtained at the 15 monitoring locations under baseline operating conditions. For model validation, inlet boundary conditions (air temperature, humidity and velocity) were set according to the control system readings of the commercial incubator during the measurement period, and the internal heat source term in the porous egg regions was adjusted such that the predicted mean temperature level was consistent with the measured values.

For each monitoring location, the simulated EST (T_sim) was extracted from the CFD results and compared with the corresponding measured value (T_meas). Model performance was evaluated by calculating the maximum absolute deviation (|T_sim − *T*_meas|_max) and the mean relative error across all monitoring points as (4-1):

(4-1) $$\text{Mean}\;\text{relative}\;\text{error}=\frac{1}{N}\sum_{j=1}^{N}|\frac{T_{\text{sim},j}-T_{\text{meas},j}}{T_{\text{meas},j}}|\times \;100\%$$

where N=15is the number of monitoring locations. Additional goodness-of-fit metrics, such as the coefficient of determination (R²) between T_sim and T_meas, were also computed.

### Structural optimization scenarios

Two structural optimization scenarios were evaluated using the validated CFD model to improve airflow distribution and temperature uniformity within the tunnel incubator. Both scenarios were designed to be simple and practically feasible in commercial hatcheries.

In Scenario 1 (transverse trolley orientation), all five egg trolleys were rotated by 90° around their vertical axis, changing the orientation of the egg trays from facing the airflow frontally (baseline) to facing it laterally. This modification altered the effective flow cross-section and the penetration of air through the egg stacks. The trolley frames and clearances to the surrounding walls were adjusted in the geometric model to reflect realistic implementation of the transverse arrangement.

In Scenario 2 (perforated flow-straightening plate), a perforated metal plate was introduced upstream of the first trolley in the airflow direction. The plate had an open area ratio of 0.26, a thickness of 2 mm and circular holes with a diameter of 10 mm, consistent with the prototype designed in the engineering part of the study. The plate extended across the full cross-section of the air tunnel, with appropriate mounting clearances to the walls.

For each scenario, the CFD model was re-run using the same boundary conditions as the baseline simulation. Trolley-level temperature non-uniformity indices (CNU, %) were re-calculated for each configuration. See detail in supplement materials.

### Field validation of Scenario 1

Based on the CFD results, Scenario 1 (transverse trolley orientation) was selected for field validation in the commercial hatchery. The physical trolleys in the same tunnel incubator were reoriented from the baseline longitudinal alignment to the transverse orientation, following the configuration used in the CFD model. No other changes were made to the incubator, control settings or hatchery routine.

After implementation of the transverse trolley orientation, ESTs were re-measured at the same 15 monitoring locations as in the baseline measurement, using the same sensors and data acquisition system. Data collection was carried out over a 12-h period under standard operating conditions. For each trolley, ΔT_max and the temperature non-uniformity index (CNU, %) were calculated as described previously. The baseline and post-modification indices are presented in Table 3, and the corresponding temperature maps are shown in Table 4, 5.

### Statistical analysis

Hatchability, mean hatch time and chick weight uniformity were analyzed using SPSS 26; IBM Corp., Armonk, NY, USA. The experimental unit for hatchability and chick weight uniformity was the trolley within each tier, based on fertile egg counts and chick weights per trolley. Tier (top, middle, bottom) was included as a fixed effect in the statistical model, and trolley within tier was included as a random effect.

Data were checked for normality and homogeneity of variances using residual plots and formal tests (Shapiro–Wilk test). When necessary, proportional data (hatchability, chick uniformity) were arcsine square root transformed prior to analysis to stabilize variances; untransformed means are presented in the tables and figures for clarity. Differences among tiers were evaluated using one-way ANOVA, followed by Tukey’s multiple comparison test to separate means when the main effect was significant. Statistical significance was declared at *p* < 0.05, and trends were discussed when 0.05 ≤ *p* < 0.10.

## RESULTS

### Temperature distribution in the commercial tunnel incubator

**Fig. 2:**
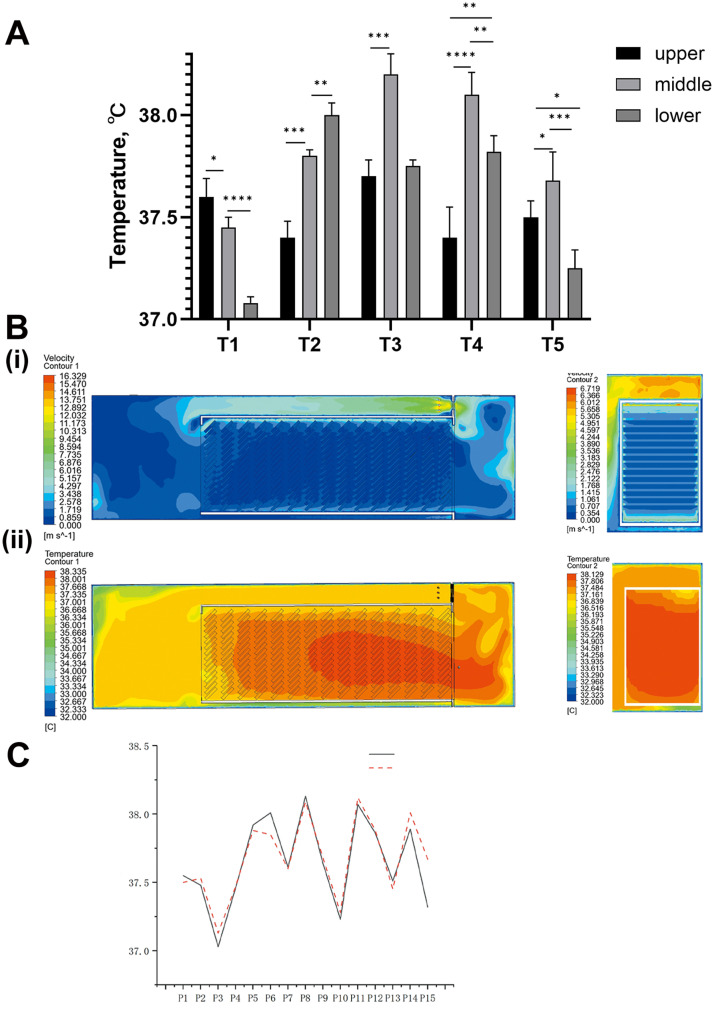
**Analysis of the baseline thermal microenvironment and model validation.** (A) Measured temperatures. Mean eggshell temperature (12-h averages) at 15 locations within the tunnel incubator. For each trolley (T1–T5), temperatures are shown for the upper, middle, and lower tray levels. Bars (or points) represent means, and error bars represent SD of the 12-h recording period. *p < 0.05, ** *p* < 0.01, *** *p* < 0.001, **** *p* < 0.0001 (B) (i) velocity contours at *Y* = 0.75 m and velocity contours at *X* = 0.5 m, (ii) temperature contours at *Y* = 0.75 m and temperature contours at *X* = 0.5 m. (C) Comparison between measured and simulated temperatures at different measurement points.

The incubator exhibited a complex temperature field characterized by significant spatial heterogeneity. Vertically, the middle tier consistently represented the hottest zone, with the exception of trolley T1. Longitudinally, trolleys T3 and T4 were identified as the primary hotspots with the highest overall temperatures, whereas the bottom of T1 appeared as a distinct cold spot (Fig. 2A, B, C). Over the 12 h monitoring period, the maximum temperature difference within a single trolley reached 0.84°C, and the maximum difference between trolleys reached 1.14°C. The average EST of the five trolleys ranged from 37.35 to 37.80°C, and the corresponding non-uniformity coefficients were 0.62%, 0.64%, 0.63%, 0.95%, and 0.63% from the inlet to the outlet side (Table 1).

### Hatchability and chick quality at different tray levels

Hatching performance differed among tray levels within the tunnel incubator (Table 2). The middle level exhibited the highest hatchability (90.63 ± 3.13%), which was significantly higher (*p* < 0.05) compared to both the upper (86.46%) and lower (83.34%) levels. Embryos in the middle level hatched significantly earlier, with a mean hatching time of 486.5 h, compared to those in the upper (487.9 h) and lower (489.07 h) levels. Furthermore, the middle level demonstrated the most synchronized hatching process, indicated by the narrowest hatch window (20.2 ± 1.5 h). In contrast, the hatch windows for the upper and lower levels were significantly prolonged to 30.5 h and 32.8 h, respectively (*p* < 0.05). Chick weight uniformity was significantly poorer in the upper level (73.03 ± 9.27%) compared to the middle (87.35%) and lower (82.80%) levels (*p* < 0.05), with no significant difference observed between the middle and lower tiers.

**Table 2:** Hatching performance of broiler chicks at different tray levels in a tunnel incubator.

Position	Hatchability (%)	Mean hatching time (h)	Hatch window (h)	Chick uniformity (%)
Upper	86.46 ± 6.50	487.90 ± 1.97	30.5 ± 2.1^a^	73.03 ± 9.27^b^
Middle	90.63 ± 3.13	486.50 ± 0.40	20.2 ± 1.5^b^	87.35 ± 2.06^a^
Lower	83.34 ± 10.04	489.07 ± 1.35	32.8 ± 3.2^a^	82.80 ± 3.87^a^

Values are presented as mean ± standard deviation (SD), *n* = 5. Different letters represent significant difference, *p* < 0.05.

### Effects of horizontal egg trolley arrangement on thermal uniformity (CFD analysis)

**Fig. 3:**
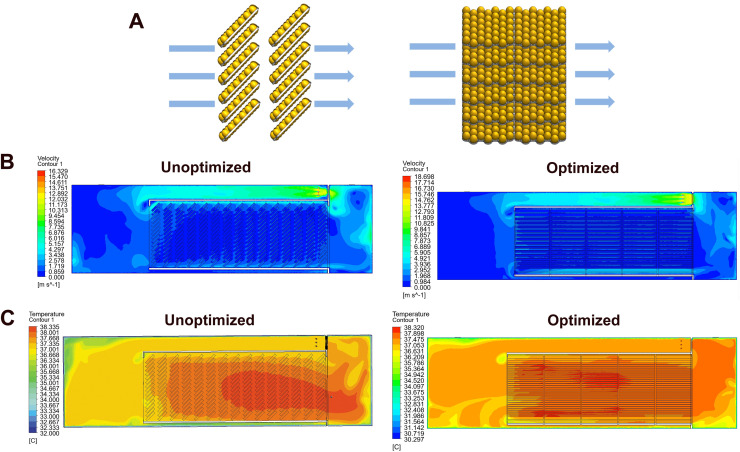
**Simulate Optimization via transverse trolley orientation.** (A) Schematic of the proposed structural modification changing trolleys from longitudinal to transverse orientation. (B) Improved airflow velocity contours and temperature fields under the optimized configuration.

In the first optimization scheme, egg trolleys were rotated 90° so that air flowed across the side of the egg trays rather than their front surfaces (Fig. 3A). CFD simulations under this “horizontal arrangement” indicated marked improvements in thermal uniformity across the five trolleys (Fig. 3B, C). Max ΔT was significantly reduced from 0.54 ± 0.21 °C in the baseline to 0.25 ± 0.09 °C after optimization (Table 3, *p* < 0.05). Correspondingly, the coefficient of non-uniformity (CNU) decreased significantly from 0.62 ± 0.33% to 0.29 ± 0.12% (*p* < 0.05), reflecting a substantial reduction in spatial temperature deviations. The mean temperature remained stable with no significant difference observed between the pre-optimization (37.68 ± 0.07 °C) and post-optimization (37.56 ± 0.06 °C) scenarios.

**Table 3:** CFD-predicted eggshell temperature parameters for trolley before and after transverse trolley orientation.

	Max ΔT (°C)	Mean T (°C)	CNU (%)
Before optimization	0.54±0.21^a^	37.68±0.07	0.62±0.33^a^
After optimization	0.25±0.09^b^	37.56±0.06	0.29±0.12^b^

CNU, coefficient of non-uniformity. Different letters represent significant difference, *p* < 0.05.

### Experimental validation of the horizontal trolley arrangement

The horizontal trolley arrangement was further validated under commercial conditions using a modified incubator in the same hatchery. Five trolleys were rotated 90° and equipped with the same miniature EST loggers used in the baseline experiment. After optimization, the measured non-uniformity coefficients of trolleys 1–5 were reduced to 0.11%, 0.36%, 0.37%, 0.35% and 0.23%, respectively (Table 4). The pattern of improvement in maximum intra-trolley temperature differences was consistent with the simulations, and the overall agreement between measured and predicted values confirmed the robustness of the CFD-guided design (Table 5)

**Table 4:** Comparison of measured and simulated values at different egg trolley positions after transverse trolley orientation in the commercial incubator (field validation).

Trolley	Max ΔT (°C)	Mean T (°C)	CNU (%)	MRE (%)
T1	0.09	0.06	0.24	0.17
T2	0.10	0.06	0.26	0.16
T3	0.04	0.02	0.10	0.07
T4	0.07	0.05	0.18	0.13
T5	0.12	0.06	0.32	0.16

Max ΔT, Mean T and CNU represent the absolute differences between measured and simulated values; MRE is the mean relative error for all monitoring points in the trolley. $MRE\;=\frac{1}{N}\setminus sum_{i=1}^{N}|\frac{T_{sim,i}-T_{meas,i}}{T_{meas,i}}|\setminus times\;100\%.$

**Table 5:** Measured eggshell temperature parameters before and after transverse trolley orientation in the commercial tunnel incubator (field validation).

	Max ΔT (°C)	Mean T (°C)	CNU (%)
Before optimization	0.6 ± 0.2^a^	37.65±0.05	0.69±0.31^a^
After optimization	0.25±0.07^b^	37.56±0.04	0.28±0.1^b^

Different letters represent significant difference, *p* < 0.05.

### Effects of installing a perforated baffle upstream of the first trolley

**Fig. 4:**
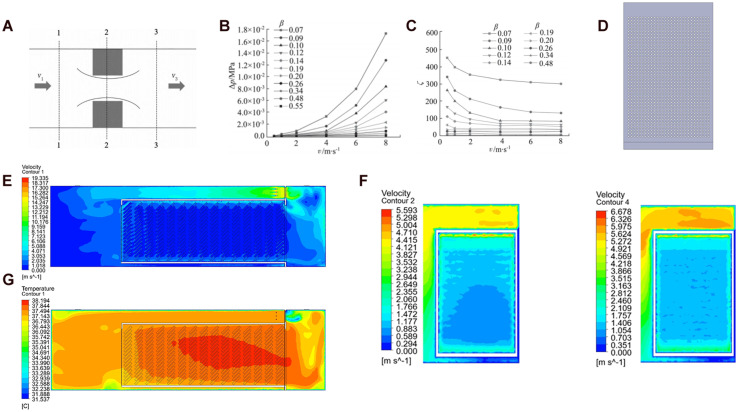
**Scheme with installation of the flow-distribution plate (CFD-only).** (A) Airflow through a single-orifice thin-plate model (B) Pressure drop–velocity relationship of the flow-distribution plate. (C) Local loss coefficient–velocity relationship for flow-distribution plates with different porosities. (D) Flow-distribution plate model. (E) Post-optimization velocity contours at *Y* = 0.75 m (F) Comparison of velocity contour plots at *X* = 1.8 m before and after optimization (G) Post-optimization temperature contours.

The second optimization scheme introduced a perforated flow-equalizing plate upstream of trolley 1. Based on resistance simulations, a perforated plate with 2-mm thickness, 10-mm hole diameter and 0.26 open area ratio was selected. CFD results showed that the baffle improved airflow distribution and reduced temperature gradients across all trolleys (Fig. 4). The optimization significantly improved the thermal uniformity within the trolley (Table 6).

**Table 6:** Comparison between measured and CFD-predicted eggshell temperature parameters for trolley after installing a perforated baffle upstream (model validation).

	Max ΔT (°C)	Mean T (°C)	CNU (%)
Before optimization	0.54±0.22^a^	37.68±0.08	0.62±0.32^a^
After optimization	0.34±0.12^b^	37.72±0.09	0.38±0.14^b^

Different letters represent significant difference, *p* < 0.05.

## Discussions

### Characterization of Baseline Thermal Non-uniformity and Airflow Mechanisms

The present study confirms that substantial temperature non-uniformity can occur inside commercial tunnel incubators, even when the overall set-point temperature is tightly controlled. Trolley-level maximum temperature differences of up to approximately 0.8°C and non-uniformity indices approaching 1% were observed under the baseline configuration. Such deviations are consistent with earlier reports showing that air velocity and temperature can vary markedly within single-stage or multi-stage incubators, particularly in large machines with complex airflow paths and high egg densities (Araújo et al., 2016; Kang et al., 2020). Although these deviations may appear small in absolute terms, eggs are highly sensitive to thermal conditions, and sustained differences of 0.5–1.0°C around the optimal temperature can meaningfully alter the rate of embryonic development (Wijnen et al., 2020).

The observed thermal heterogeneity, characterized by a stable middle tier and variable top and bottom tiers, results from the combined effects of forced convection, buoyancy, and local flow obstructions. Warm air supplied from the air handling unit tends to rise and recirculate, whereas dense egg stacks and trolley frames create preferential paths and dead zones. Similar mechanisms have been identified in CFD analyses of cabinet-type incubators and smaller experimental units (N., Pourvosoghi, 2018; Küçüktopcu and Cemek, 2019), but quantitative field data for commercial tunnel incubators have been scarce. By providing both measured and simulated temperature distributions at the egg level, the present work bridges this gap and demonstrates that commercial tunnel incubators may operate with a narrower safety margin than hatchery managers might expect based solely on cabinet sensor readings. From a hatchery management perspective, the observed non-uniformity implies that eggs placed in different regions of the same machine effectively experience different incubation programs, even when set-point conditions are identical. This heterogeneity complicates interpretation of overall hatch performance and makes it difficult to diagnose subtle problems in specific trolleys or tiers. The findings therefore highlight the importance of moving beyond chamber-level temperature control and considering egg-level thermal conditions when assessing incubator performance and designing corrective actions.

### Influence of Vertical Position on Hatching Performance and Chick Quality

One of the key findings of this study is that vertical position within the tunnel incubator had a clear and consistent impact on hatchability, hatch timing and chick uniformity. Eggs located on the middle tier, where mean EST was closer to the target and less variable, achieved the highest hatchability, the shortest and most synchronized hatch window and the greatest chick weight uniformity. In contrast, eggs on the top and especially the bottom tiers showed reduced hatchability, delayed and more dispersed hatching and poorer uniformity. These observations align with the well-established principle that deviations from the optimal temperature slow or accelerate embryonic development and increase the risk of mortality (Boerjan, 2006; Mafruchati et al., 2023). Experimental studies in cabinet incubators have demonstrated that sustained reductions in EST (e.g., from 37.8 to 37.0°C) can prolong incubation time and increase late embryo mortality, whereas higher temperatures can cause early hatching and increased incidence of unviable chicks(Lourens et al., 2005). Moreover, non-uniform temperatures within a batch lead to expand the hatch window, which has been associated with impaired yolk sac resorption, reduced chick quality scores (dehydration or unhealed navels), and compromised early broiler performance (Bergoug et al., 2013; Jabbar et al., 2020). The present results extend these findings to the context of a commercial tunnel incubator and show that vertical gradients within a single machine can produce biologically meaningful differences in hatch performance. The fact that the bottom tier did not consistently perform better than the top tier, despite being closer to the floor, suggests that temperature variability and local airflow patterns, rather than absolute elevation alone, drive the observed differences. In the present tunnel incubator, the bottom tier experienced both cooler and more fluctuating conditions, likely due to interaction between the main airflow and returning air currents. This may explain why bottom-tier hatchability and chick uniformity were often inferior to those of the middle tier and sometimes comparable to or worse than those of the top tier. These results underscore the need to consider both mean temperature and its stability when interpreting vertical position effects.

From a practical standpoint, the vertical differences observed in this study imply that hatchery managers should be cautious when assigning eggs of different genetic lines, breeder ages or flock health status to different regions of a tunnel incubator. Placing more sensitive or high-value eggs preferentially on the most favorable tiers (e.g., middle) may partially mitigate risks associated with non-uniform conditions. However, such strategies are only an interim solution; ultimately, the goal should be to improve the uniformity of the entire machine through engineering and operational interventions, as explored in this work.

### Insights from CFD Modeling: Validation and Airflow Diagnostics

The use of CFD in the present study provided valuable insights into the internal airflow and temperature fields that are difficult or impossible to obtain from measurements alone. By treating the egg stacks as porous media and validating the model against measured ESTs, we achieved good agreement between simulated and measured temperatures. These error levels are comparable to, or lower than, those reported in validated CFD studies of poultry incubators/egg setters, where model–measurement agreement for temperature and key scalars is typically within a few percent, and can remain within ∼10–12% depending on the variable and sensor coverage(Fernandes et al., 2026; SASE et al., 2009). Importantly, the CFD results not only reproduced the observed temperature gradients but also revealed the underlying airflow structures, such as jets, recirculation zones and high-resistance regions caused by trolley geometry and egg stacks. This information is critical for understanding why certain trolleys or tiers perform poorly and for designing targeted modifications. For example, the baseline simulation showed that airflow in the present tunnel incubator tended to bypass parts of the egg stacks, creating hot and cold spots and contributing to the elevated non-uniformity indices. CFD has been increasingly used to study incubator designs, including optimization of fan placement, vent openings and control strategies. However, many published models are based on idealized geometries or lack thorough validation, which can limit their practical applicability. The present study demonstrates a workflow that may be particularly useful for commercial hatcheries: (i) obtain egg-level temperature measurements in operating machines; (ii) develop a porous-medium CFD model that reproduces these measurements with acceptable accuracy; and (iii) use the model to test structural or operational modifications before implementing them in the field. This approach balances computational efficiency with biological relevance and helps ensure that proposed interventions are both technically sound and economically feasible.

### Evaluation and field validation of structural optimization strategies

Both structural optimization scenarios evaluated in this study significantly improved modelled temperature uniformity compared with the baseline configuration. Rotating the trolleys to a transverse orientation (Scenario 1) increased airflow penetration through the egg stacks, reduced recirculation zones and lowered trolley-level non-uniformity indices to as little as 0.10–0.43% in the CFD simulations. Installing a perforated flow-straightening plate upstream of the first trolley (Scenario 2) also improved airflow distribution and reduced non-uniformity (0.34–0.46%), primarily by homogenizing inlet airflow and attenuating longitudinal temperature gradients. Although both scenarios were beneficial, Scenario 1 generally achieved the lowest non-uniformity indices across trolleys, particularly in the central region of the incubator. Crucially, Scenario 1 was further validated under commercial conditions. After reorienting the trolleys in the actual tunnel incubator, measured non-uniformity indices decreased from 0.62 to 0.95% to 0.11–0.37%, and the EST maps became visibly more homogeneous. The magnitude and pattern of improvement closely matched the CFD predictions, providing strong evidence that this structural modification is both effective and practically feasible. From a hatchery standpoint, transverse trolley orientation has the advantage of requiring no additional components, minimal capital investment and relatively simple implementation, especially in new installations or when trolleys are being replaced.

The perforated plate approach (Scenario 2) may be attractive in situations where trolley orientation cannot be easily changed, or where further fine-tuning of airflow is desired. However, such devices may increase system pressure drop, potentially affecting fan performance and energy consumption if not properly accounted for. Detailed assessments of energy use, maintenance requirements and long-term durability would be needed before widespread adoption. In contrast, the results of the present study suggest that simply reorienting trolleys, where structurally feasible, can yield substantial benefits with minimal downside.

Although we did not directly measure the impact of these structural modifications on hatchability and chick quality in this study, improvements in temperature uniformity of the magnitude observed here would be expected to narrow the hatch window and enhance chick uniformity and the tier effects documented under the baseline configuration. Future work should explicitly quantify these performance outcomes over multiple hatches and economic cycles to provide a more comprehensive cost–benefit analysis for hatcheries.

### Study limitations and future perspectives

Several limitations of the present study should be acknowledged. First, all measurements and trials were conducted in a single commercial tunnel incubator and hatchery, using eggs from one breeder flock. Although this approach ensured good control of confounding factors and reflects a realistic commercial scenario, it limits the generalizability of the findings to other incubator models, hatchery designs and genetic lines. Replication of the study in multiple hatcheries and under different loading conditions would strengthen the evidence base and help determine how widely applicable the structural optimization strategies are. Second, we focused primarily on temperature as the key environmental variable, and we did not characterize spatial variations in relative humidity, CO₂ concentration or air velocity at the egg level. These factors can also influence embryonic development and chick quality (Adame and Ameha, 2023), and they may interact with temperature in complex ways. Integrating multi-parameter monitoring (e.g., temperature, humidity, CO₂) with CFD models that include moisture and gas transport would provide a more complete picture of the incubator microenvironment. Third, hatch performance was assessed at the end of incubation in terms of hatchability, mean hatch time and chick weight uniformity, but we did not follow the chicks into the broiler grow-out phase. Previous studies have shown that differences in incubation conditions can have lasting effects on post-hatch growth, feed efficiency, immune function and carcass traits (Molenaar et al., 2010). Longitudinal studies linking egg-level thermal conditions and structural modifications to broiler performance throughout the production chain would therefore be valuable. Finally, the porous-medium representation of egg stacks, while computationally efficient and sufficiently accurate for thermal predictions in this study, inevitably simplifies the complex geometry of individual eggs and trays. More detailed models resolving individual eggs and including transient embryonic heat production could offer additional insights into local-scale phenomena, such as the effects of egg size distribution or tray design. However, such models are computationally demanding and may be less practical for routine hatchery applications.

Despite these limitations, the present study demonstrates that combining field measurements, CFD analysis and practical structural modifications can provide a powerful framework for diagnosing and improving the environmental conditions in commercial tunnel incubators. Future research should build on this approach to develop and validate additional engineering and management strategies aimed at enhancing hatchability, chick quality and overall efficiency in the poultry industry

## Conclusions

This study confirms that significant vertical temperature gradients exist within commercial single-stage tunnel incubators, which directly compromise hatchability and chick uniformity in the upper and lower tiers. Through validated CFD modeling and field experiments, we demonstrated that the standard longitudinal trolley arrangement creates inherent airflow resistance that exacerbates these thermal deviations. Crucially, the implementation of a transverse trolley orientation proved to be an effective, low-cost engineering solution that substantially homogenizes the thermal microenvironment. These findings provide hatcheries with a practical strategy to mitigate spatial non-uniformity, thereby enhancing overall flock quality and production efficiency without requiring complex equipment retrofits.

## CRediT authorship contribution statement

**Mingyang Li:** Writing – review & editing, Writing – original draft, Visualization, Methodology, Formal analysis, Data curation. **Zefeng Shi:** Formal analysis, Data curation. **Zongchun Bai:** Resources. **Yinong Hu:** Software, Resources. **Guofeng Han:** Writing – review & editing, Supervision, Project administration, Funding acquisition, Conceptualization.

## Disclosures

The authors declare that they have no known competing financial interests or personal relationships that could have appeared to influence the work reported in this paper.

## Appendix Supplementary materials

Image, application 1

## Footnotes

Supplementary material associated with this article can be found, in the online version, at doi:10.1016/j.psj.2026.106666.
