# Supplementary material for: Egg-level temperature monitoring and CFD-guided structural optimization improve thermal uniformity and hatch performance in a single-stage tunnel incubator
Source: Poult Sci. 2026 Feb 18;105(5):106666. doi: 10.1016/j.psj.2026.106666 (PMC12945628; doi:10.1016/j.psj.2026.106666)
Supplement: Supplementary file 1 [file mmc1.docx]

**Fundamental governing equations of computational fluid dynamics**

The theoretical basis for constructing the governing equations of fluid flow in CFD originates from the fundamental conservation laws of fluid motion. These laws comprise the conservation of mass, momentum and energy, and form the core theoretical framework for describing fluid-flow characteristics. In CFD numerical simulations, these three types of governing equations are typically discretized and solved, thereby yielding algebraic systems from which the spatial distributions of pressure, velocity, temperature and other physical quantities in the flow field can be obtained. When the fluid system involves multicomponent mixtures, additional species conservation equations must be introduced.
In this study, we focus solely on the distribution characteristics of air temperature inside the incubator, and therefore do not consider species conservation. During the solution procedure, a closed set of governing equations is constructed by simultaneously solving the mass, momentum and energy conservation equations, ensuring that the numerical computation can accurately capture the coupling between the flow field and the temperature field and provide a reliable theoretical basis for the subsequent simulation analysis.

**Mass conservation equation**

In CFD numerical simulations, the fluid medium is usually assumed to satisfy the continuum hypothesis, namely that fluid particles are continuously distributed in space within the flow field. The essence of the mass conservation law is that, for a differential control volume, the rate of mass inflow per unit time must balance the rate of mass accumulation within the control volume. On this basis, the mass conservation equation applicable to fluid-flow problems can be derived

$\frac{\partial(\rho u)}{\partial x}+\frac{\partial(\rho v)}{\partial y}+\frac{\partial(\rho w)}{\partial z}=0$ (3-1)

where $\rho$is the fluid density (kg/m^3^), and u、v、w are the velocity components in the x, y and z directions, respectively (m/s).

**Momentum conservation equations**

The momentum conservation law is a fundamental principle that any flow system must satisfy. It states that the rate of change of fluid momentum within a differential control volume with respect to time is equal to the sum of all external forces acting on that control volume. According to the momentum conservation law, the momentum equations in the x, y and z directions can be written as:

$\frac{\partial(\rho u)}{\partial t}+div\left( \rho uu \right)=-\frac{\partial\rho}{\partial x}+\frac{\partial\tau_{xx}}{\partial x}+\frac{\partial\tau_{yx}}{\partial y}+\frac{\partial\tau_{zx}}{\partial z}+F_{x}$ (3-2a)

$\frac{\partial(\rho v)}{\partial t}+div\left( \rho vu \right)=-\frac{\partial\rho}{\partial y}+\frac{\partial\tau_{xy}}{\partial x}+\frac{\partial\tau_{yy}}{\partial y}+\frac{\partial\tau_{zy}}{\partial z}+F_{y}$ (3-2b)

$\frac{\partial(\rho w)}{\partial t}+div\left( \rho wu \right)=-\frac{\partial\rho}{\partial z}+\frac{\partial\tau_{xz}}{\partial x}+\frac{\partial\tau_{yz}}{\partial y}+\frac{\partial\tau_{zz}}{\partial z}+F_{z}$ (3-2c)

where $\rho$ is the pressure acting on the fluid microelement; $\tau_{xx}$、$\tau_{xy}$, $\tau_{xz}$ etc. are components of the viscous stress tensor$\tau$ on the surface of the microelement arising from molecular viscosity; and$F_{x}$、$F_{y} F_{z}$are the components of body force per unit volume. If gravity is the only body force and the z-axis is oriented vertically upward, then $F_{x}=0$，$F_{y}=0$，$F_{z}=\rho g$

**Energy conservation equation**

The energy conservation law is the fundamental principle describing heat exchange and transfer during fluid flow. Its core concept is that the rate of change of internal energy within a differential control volume per unit time is equal to the sum of the work done by external body forces and the net heat input. Based on this law, the energy conservation equation can be derived:

$\frac{\partial(\rho T)}{\partial t}+div\left( \rho uT \right)=div\left[ \frac{k}{c_{p}}gradT \right]+S_{T}$ (3-3)

Written in expanded form:$\frac{\partial\left( \rho T \right)}{\partial t}+\frac{\partial\left( \rho uT \right)}{\partial x}+\frac{\partial\left( \rho vT \right)}{\partial y}+\frac{\partial\left( \rho wT \right)}{\partial z}$

$=\frac{\partial}{\partial x}\left( \frac{k}{c_{p}}\frac{\partial T}{\partial x} \right)+\frac{\partial}{\partial y}\left( \frac{k}{c_{p}}\frac{\partial T}{\partial y} \right)+\frac{\partial}{\partial z}\left( \frac{k}{c_{p}}\frac{\partial T}{\partial z} \right)+S_{T}$ (3-4)

where $c_{p}$ is the specific heat at constant pressure, T is the thermodynamic temperature, k is the thermal conductivity of the fluid, and $S_{T}$ is the viscous dissipation term. Equations (3-3) and (3-4) are often collectively referred to as the energy equation.

**Establishment of the porous-media model of the egg tray**

In practical production, egg trays are not completely closed; warm air inside the incubator flows through the small holes in the egg trays and carries away a portion of the heat. When using CFD to simulate environmental parameters in the incubator, the characteristic size of these small holes is much smaller than the overall dimensions of the incubator. Explicitly resolving all these small holes in the computational mesh would lead to a dramatic increase in cell count and a deterioration of mesh quality. If the holes in the egg tray are ignored during model simplification, the discrepancy between simulation and actual measurements becomes large. Thus, the presence of the holes must be considered during modelling to reduce error.
Wu et al. simplified slatted floors and pig activity areas using a porous-media model when simulating ventilation in nursery pig houses, thereby reducing the number of mesh elements. Therefore, to reduce computational cost while improving numerical accuracy, the egg tray is simplified as a porous medium in this study for the purposes of numerical simulation.

**Porous-media model**

In CFD, porous-media models are commonly used to handle flow problems in regions with complex geometrical or porous structures, especially where direct mesh generation is difficult. Essentially, this model introduces an additional momentum source term into the momentum equations, consisting of a viscous resistance loss term and an inertial resistance loss term.
In ANSYS Fluent, flow calculations in porous regions are typically carried out based on the superficial velocity, and the pressure-drop characteristics of the medium are described by the Darcy–Forchheimer equation:

$\frac{\Delta P}{\Delta n}=-\left( D\mu\nu_{n}+C\frac{1}{2}\rho\left| \nu\right|\nu_{n} \right)$ (3-5)

where P is the fluid pressure (Pa); n is the distance along the flow direction (m); μ is the dynamic viscosity (Pa·s); D is the viscous resistance coefficient m^-2^; C is the inertial resistance coefficient m^-1^; $\rho$ is the fluid density kg/m^3^; and $\nu$ is the velocity vector (m/s), with $\nu$ being its component in the flow direction.

According to this equation, the relationship between unit pressure drop and flow velocity is a quadratic function. In the porous-media model calculations, once the relationship between unit pressure drop and flow velocity is known, the key parameters D and C can be obtained by curve fitting.

**Physical model and computational domain of the egg tray**

A 1:1 scale three-dimensional model of the egg tray was constructed in SolidWorks, with 42 hatching eggs placed in each tray.
Each layer of the egg trolley consists of four egg trays that are tightly fitted together. Therefore, in simplifying the porous-media model, four tightly packed egg trays can be regarded as a single unit. Because airflow can pass through the egg tray in the X, Y and Z directions, the resistance coefficients in all three directions must be determined. The overall dimensions of the computational domain are 1190 mm in length, 300 mm in width and 60 mm in height.

**Mesh generation and boundary conditions**

A polyhedral mesh was generated for the egg-tray computational domain. To ensure numerical convergence, local mesh refinement was applied to the surfaces of the egg tray, resulting in a total of 150,000 cells with good mesh quality. The working fluid was air. The inlet was set as a velocity inlet with velocity values of 0.1, 0.2, 0.3, 0.4, 0.5, 0.6, 0.7, 0.8, 0.9 and 1.0 m/s, and the outlet was set as a pressure outlet.

First, the relationship between unit pressure drop and velocity in the X direction was calculated. The pressure drops across the egg tray at different airflow velocities were obtained from the simulations, and the resulting data for unit pressure drop and velocity were fitted using a quadratic function. The fitted expression for the unit pressure drop （$\frac{\Delta P}{\Delta x}$）$\Delta v$ as a function of velocity $\Delta v$ was: $\frac{\Delta P}{\Delta x}=49.988v_{x}^{2}+7.757v_{x}$*.*

Based on this equation, theoretical calculations yielded a viscous resistance coefficient D of 434,468.466 m^-2^ and an inertial resistance coefficient C of 81.613 m^-1^ for the X direction.

Similarly, the fitted equation in the Y direction was

$$\frac{\Delta P}{\Delta y}=34.874v_{y}^{2}+6.279v_{y}$$

from which a viscous resistance coefficient D of 351,685.897 m^-2^ and an inertial resistance coefficient C of 56.937 m^-1^ were obtained for the Y direction.

In the Z direction, the fitted equation was:

$$\frac{\Delta P}{\Delta z}=1477.116v_{z}^{2}+34.89v_{z}$$

and the corresponding viscous resistance coefficient D and inertial resistance coefficient C were calculated to be 1,954,183.936 m^-2^ and 2411.618 m^-1^, respectively.

These viscous resistance coefficients D and inertial resistance coefficients C for the three directions were then input into the porous-media model so that the unit pressure drop would match that of the actual egg-tray model. In this way, the porous-media model can effectively replace the real egg-tray structure and reproduce the same hydrodynamic characteristics.

**Numerical simulation of the incubator internal environment**

**Physical model of the incubator and computational domain**

The overall dimensions of the incubator and the internal egg trolleys were measured with a tape measure and used to construct the physical model. To streamline the modelling process, improve computational efficiency and shorten solution time, the incubator model was reasonably simplified as follows:

(1) In the actual tunnel-type incubator, guide rails are installed at the bottom to facilitate movement of the egg trolleys and to position each trolley. Because their influence on internal air circulation is minimal, the guide rails were omitted in the model.

(2) In practice, the egg trolley is not shielded on any side. However, during incubation, the left side of the trolley is in close contact with the inner wall of the incubator, and a baffle is installed on the right side to prevent airflow from escaping from the sides. Accordingly, in the model the two sides of the trolley were simplified as closed boundaries.

(3) The water pan inside the incubator is located beneath the fan and above the top of the egg trolley. Its influence on the internal airflow is minor, and therefore it was omitted in the model.

(4) During incubation, a large number of hatching eggs are present in the incubator, and there are many small holes in the egg trays. If the egg trays and eggs were not simplified, the mesh count would be extremely high, making the calculations very difficult. Conversely, if the small holes in the egg trays were ignored, the simulation results would be inaccurate. Therefore, the egg trays and eggs were treated as porous media. The viscous and inertial resistance coefficients of the egg trays and eggs were calculated, and the porous regions were represented by cubic fluid domains in the model.

Because the internal structure of the incubator is symmetric, the model was cut along the central symmetry plane to reduce computational cost, and this plane was set as a symmetry boundary. After extracting the fluid domain, the resulting model represents one half of the incubator.

**Model processing and mesh generation**

In CFD simulations, mesh size is a key factor affecting computational efficiency and resolution of the flow field, while mesh quality directly determines the accuracy of the results and the convergence behaviour. In the present work, Poly-Hexcore meshing technology was used in the mesh generation process, enabling node-to-node coupling between structured hexahedral cells and unstructured polyhedral cells. This mesh type increases the proportion of hexahedral cells, thereby improving computational accuracy, solution efficiency and convergence, and making the numerical results more reliable.

A base cell size of 50 mm was applied to the overall incubator domain. Because the heating elements are much smaller than the overall incubator size, local mesh refinement with a cell size of 8 mm was applied around the heating elements to ensure mesh quality. Mesh statistics showed that the final mesh contained 485,680 nodes and 1,979,924 cells.

Mesh quality strongly influences solution efficiency, convergence and result accuracy; therefore, a quality assessment was conducted after meshing to ensure computational reliability. Cell quality was used as the evaluation metric. The computed average cell quality was 0.825, indicating that the overall mesh quality was good and met the accuracy requirements for subsequent numerical solutions.

**Boundary-condition settings**

The procedure for setting boundary conditions was as follows: first, all boundary surfaces were defined in the meshing tool; then, corresponding boundary types (velocity inlet, pressure outlet) were assigned in Fluent. Experimental measurements of airflow velocity and pressure in the incubator were used to specify key parameters and ensure physically reasonable boundary conditions. This process encompasses the definition of operating conditions, fluid material properties and parameterization of flow boundaries.

(1) Selection of turbulence model

The standard k–ε turbulence model is widely used in numerical simulations related to poultry production because of its low computational cost and modest hardware requirements, and it is one of the most common simplified models for such problems. Therefore, in all simulations in this study, the Realizable k–ε model was selected as the turbulence model.

(2) Determination of simulation regime

For a fully enclosed incubator, the airflow can be considered to have reached a steady state after the fan has been operating for a sufficiently long period. Accordingly, steady-state simulations were adopted to represent the internal environment of the incubator and to more accurately reflect the actual operating conditions.

(3) Basic fluid settings
The working fluid inside the incubator was set as air, which was assumed to be an incompressible ideal gas. Gravitational acceleration of 9.81 m/s was applied in the negative Z direction.

(4) Boundary-condition settings
The inlet was specified as a velocity inlet. Based on measured fan velocity in the incubator, the inlet velocity was set to 14 m/s and the temperature to 37.3 °C. The outlet was defined as a pressure outlet. The heating elements were modelled as volumetric energy sources with an energy source term of 8100 W/m^3^. Considering that hatching eggs are also heat sources during incubation, different heat-generation rates were assigned to the eggs at different incubation days according to the results of M. NICHELMANN et al. The inner walls of the incubator were set.

**Numerical solution**

A three-dimensional numerical model of the flow field inside the incubator was established using ANSYS Fluent. A pressure–velocity coupling strategy was employed for iterative calculations. The SIMPLE algorithm was used as the solver. Spatial discretization of the governing equations was carried out using the finite volume method. During the computations, residuals of the velocity components, energy equation and continuity equation, as well as turbulence dissipation rate and net mass flow imbalance between inlet and outlet, were monitored in real time.

The convergence criterion for all governing equations was set to a residual level of 10^-4^. When the residuals of all physical quantities had stably fallen below 10^-4^ and the net mass flow between inlet and outlet approached zero, the solution was deemed converged and the iterative calculation was terminated.

### **Installation scheme of the flow-straightening plate**

Analysis of the internal optimization scheme of the incubator

he working fluid inside the incubator is air. As the airflow passes through the flow-straightening plate, jets are formed by the perforations, thereby achieving a uniform distribution of the flow field. These jets gradually merge in the downstream region and, after forming a more uniform flow field, discharge from the outlet. Because the resistance characteristics of the flow-straightening plate are closely related to the flow behaviour of a single orifice, a theoretical analysis of the flow through a single orifice is required. For a thin perforated plate, the internal flow can be simplified and studied using a typical contraction–expansion flow model.

When the airflow passes through a small orifice, the flow process consists of two key stages: a sudden contraction and a sudden expansion. The main flow cross-section first contracts and then expands, accompanied by dynamic changes in velocity and effective flow area, thereby generating a local resistance loss.

Neglecting the frictional loss along the flow path inside the hole, the Bernoulli equation for real fluids gives:

$\rho gz_{2}+p_{2}+\frac{1}{2}\alpha_{2}\rho v_{2}=\rho gz_{3}+p_{3}+\frac{1}{2}\alpha_{3}\rho v_{3}+\frac{1}{2}\zeta_{0}\rho v_{2}$ (4-1)

where $z_{2}$，$z_{3}$,are the elevations of sections 2–2 and 3–3, respectively; $p_{2}$，$p_{3}$ are the pressures at sections 2–2 and 3–3, respectively; $\alpha_{2}$，$\alpha_{3}$ are the kinetic-energy correction coefficients at sections 2–2 and 3–3, respectively. Because the orifice plate is installed horizontally, $\alpha_{2}=\alpha_{3}=1$. $v_{2}$，$v_{3}$are the mean velocities at sections 2–2 and 3–3, respectively; $\zeta_{0}$ is the local loss coefficient of the orifice; $\rho$ is the fluid density; and $g$ is the gravitational acceleration.

Neglecting body forces, the momentum equation yields:

$p_{2}A_{2}-p_{3}A_{3}=\rho(A_{2}v_{2}^{2}-A_{3}v_{3}^{2})$ (4-2)

where $A_{2}$，$A_{3}$ are the cross-sectional areas of sections 2–2 and 3–3, respectively.

From the continuity equation:

$A_{h}v_{h}=A_{2}v_{2}=A_{3}v_{3}$ (4-3)

where $A_{h}$is the cross-sectional area of the orifice, and $v_{h}$ is the mean velocity of the fluid within the orifice.

Combining the above equations, the expression for the local resistance coefficient of a single orifice can be obtained:

$\zeta_{0}=\frac{p_{1}-p_{3}}{2\rho v^{2}}={(\frac{A_{3}}{A_{h}}\times\frac{1}{\varepsilon}-1)}^{2}$ (4-4)

where$\zeta_{0}$ is the local resistance coefficient of a single hole in the perforated plate; $p_{1}$is the pressure at section 1–1; and$\varepsilon$is the contraction coefficient of the orifice, defined as the ratio of the minimum jet cross-sectional area to the orifice cross-sectional area, i.e. $\varepsilon=\frac{A_{2}}{A_{h}}$ depends only on the flow regime and on the geometry of the orifice.

A standard k-ε two-equation turbulence model was adopted, with no-slip wall boundary conditions imposed. Air was taken as the fluid medium in the duct. The inlet boundary condition was a velocity inlet, with the inlet velocity ranging from 0.2 to 5 m/s; the outlet boundary condition was a pressure outlet, with a gauge pressure of 0. The local resistance coefficient $\zeta$ of the flow-straightening plate was defined by equation (4-5):

$\zeta=\frac{2\times{10}^{-6}\Delta p}{\rho\nu^{2}}$ (4-5)

where $\Delta p$ is the pressure difference across the perforated plate in the steady-state region; $\rho$ is the air density; and $\nu$ is the mean flow velocity in the channel.

***
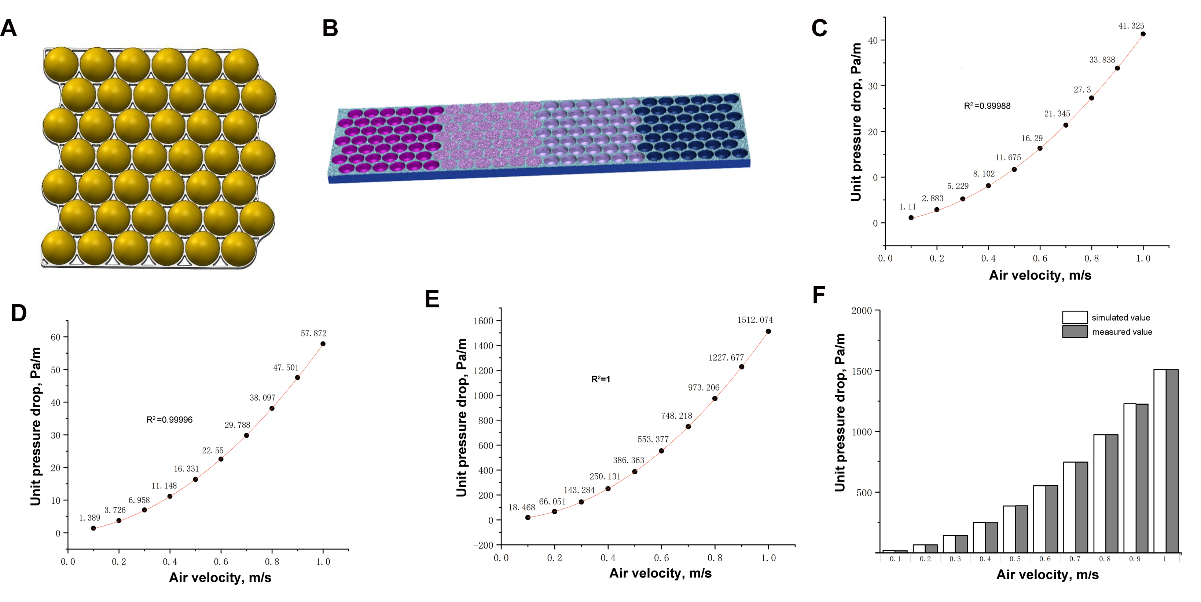
***

**Figure S1. Establishment of the porous-media model of egg trays**

(A) Egg-tray model (B) Computational domain of the egg tray (C) Unit pressure drop–velocity relationship in the X direction (egg tray) (D) Unit pressure drop–velocity relationship in the Y direction (E) Unit pressure drop–velocity relationship in the Z direction (F) Verification and simulation values of unit pressure drop of egg tray in the z-direction
